# Comparison of machine learning techniques in prediction of mortality following cardiac surgery: analysis of over 220 000 patients from a large national database

**DOI:** 10.1093/ejcts/ezad183

**Published:** 2023-05-08

**Authors:** Shubhra Sinha, Tim Dong, Arnaldo Dimagli, Hunaid A Vohra, Chris Holmes, Umberto Benedetto, Gianni D Angelini

**Affiliations:** Division of Cardiac Surgery, Bristol Heart Institute, Translational Health Sciences, University of Bristol, Bristol, UK; Division of Cardiac Surgery, Bristol Heart Institute, Translational Health Sciences, University of Bristol, Bristol, UK; Division of Cardiac Surgery, Bristol Heart Institute, Translational Health Sciences, University of Bristol, Bristol, UK; Division of Cardiac Surgery, Bristol Heart Institute, Translational Health Sciences, University of Bristol, Bristol, UK; Alan Turing Institute, London, UK; Department of Statistics, University of Oxford, Oxford, UK; Division of Cardiac Surgery, Bristol Heart Institute, Translational Health Sciences, University of Bristol, Bristol, UK; Division of Cardiac Surgery, Bristol Heart Institute, Translational Health Sciences, University of Bristol, Bristol, UK

**Keywords:** Machine learning, Mortality prediction, Cardiac surgery, Risk stratification, Benchmarking

## Abstract

**OBJECTIVES:**

To perform a systematic comparison of in-hospital mortality risk prediction post-cardiac surgery, between the predominant scoring system—European System for Cardiac Operative Risk Evaluation (EuroSCORE) II, logistic regression (LR) retrained on the same variables and alternative machine learning techniques (ML)—random forest (RF), neural networks (NN), XGBoost and weighted support vector machine.

**METHODS:**

Retrospective analyses of prospectively routinely collected data on adult patients undergoing cardiac surgery in the UK from January 2012 to March 2019. Data were temporally split 70:30 into training and validation subsets. Mortality prediction models were created using the 18 variables of EuroSCORE II. Comparisons of discrimination, calibration and clinical utility were then conducted. Changes in model performance, variable-importance over time and hospital/operation-based model performance were also reviewed.

**RESULTS:**

Of the 227 087 adults who underwent cardiac surgery during the study period, there were 6258 deaths (2.76%). In the testing cohort, there was an improvement in discrimination [XGBoost (95% confidence interval (CI) area under the receiver operator curve (AUC), 0.834–0.834, F1 score, 0.276–0.280) and RF (95% CI AUC, 0.833–0.834, F1, 0.277–0.281)] compared with EuroSCORE II (95% CI AUC, 0.817–0.818, F1, 0.243–0.245). There was no significant improvement in calibration with ML and retrained-LR compared to EuroSCORE II. However, EuroSCORE II overestimated risk across all deciles of risk and over time. The calibration drift was lowest in NN, XGBoost and RF compared with EuroSCORE II. Decision curve analysis showed XGBoost and RF to have greater net benefit than EuroSCORE II.

**CONCLUSIONS:**

ML techniques showed some statistical improvements over retrained-LR and EuroSCORE II. The clinical impact of this improvement is modest at present. However the incorporation of additional risk factors in future studies may improve upon these findings and warrants further study.

**Figure:**
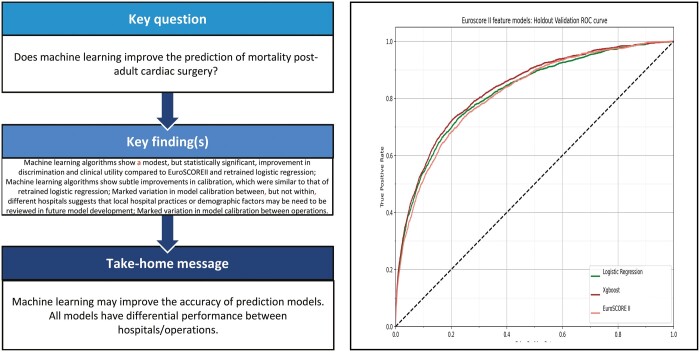

## INTRODUCTION

The inherent mortality risk associated with cardiac surgery needs to be accurately quantified using an updated and accurate risk prediction model. With the emergence of alternative treatment strategies and improving outcomes, this would allow appropriate patient counselling, provide a context for benchmarking results and form the basis of treatment guidelines.

The predominant in-hospital mortality risk stratification tool utilized in adult cardiac surgery in the UK is the European System for Cardiac Operative Risk Evaluation (EuroSCORE) II [1]—formulated in 2011. It assessed results from multiple European centres using logistic regression (LR) and utilizes 18 perioperative factors. The data relevant to its calculation are now routinely gathered, analysed and released for the above purposes. EuroSCORE II improved upon the results of logistic EuroSCORE [2] but still has a tendency to over-estimate risk, especially in those in the highest risk strata [3–8] and in those operated on outside Europe [9].

LR is a powerful tool that has been utilized for many years in the development of prediction models. It relies on key assumptions regarding the linearity of the relationship between the logit of the explanatory variables and the response variable and the lack of multi-collinearity between explanatory variables [10]. Any such complex interactions would have to be accounted for by the model developer and could decrease the final model’s performance. Machine learning (ML) can be defined as ‘the science of getting computers to learn, without being explicitly programmed’ [11]. Interest in ML-based prediction modelling has increased considerably in recent years [12, 13]. ML incorporates a multitude of different algorithms and has the advantages of not making assumptions regarding linearity and using self-generating algorithms to interpret complex variable interactions and handle large volumes of data. These techniques have limitations when handling small volumes of data or large class imbalances. Comparisons between these techniques as applied to prediction modelling in cardiac surgery has been limited [12–15]. A recent meta-analysis by our group [14] has shown some advantages of ML algorithms over LR. However, the true clinical impact of these differences was difficult to establish. Our single-centre study found no significant differences in the performance of models developed using ML compared to LR [16]. With this study, we applied similar techniques on a much larger national dataset of over 220 000 patients to investigate differences in predictive performance between EuroSCORE II, ML-based or LR-based models retrained on the EuroSCORE II variables. We perform a thorough analysis using measures of discrimination, calibration and clinical utility. Finally, we reviewed model performance over time, between hospitals, between operations and temporal changes in variables importance. We are adding to the knowledge behind model development and assessment with our paper.

## METHODS

### Ethics statement

The register-based cohort study is part of research approved by the Health Research Authority (HRA) and Health and Care Research Wales and a need for patients’ consent was waived (HCRW) (IRAS ID: 257758, 23 July 2019). Reporting of results follows the TRIPOD statement.

### Data extraction

A complete extract of prospectively collected data from the National Adult Cardiac Surgery Audit was obtained from the National Institute of Cardiovascular Outcomes Research central cardiac database of all adults undergoing cardiac surgery in England and Wales. Data processing and imputation of missing data were as previously described [17, 18] using R v4.0.2 [19]. Age and weight were imputed using the median. The absence of other variables was presumed to be an absence of that risk factor, a method previously established by National Adult Cardiac Surgery Audit. The variables unique to EuroSCORE II (e.g. Creatinine Clearance) were not routinely collected in the UK till after its publication in 2012. Hence, on analysis of the national data, we found that a meaningful comparison with EuroSCORE II could only be formulated by utilizing data from 2012 to 2019.

### Statistical analysis

Categorical variables were summarized as counts and percentages. Continuous variables were summarized as mean and standard deviation. The primary outcomes were discrimination, calibration and clinical utility of the different models in prediction of mortality risk either in-hospital or within 30 days of cardiac surgery. Comparisons of quantitative model performance were performed using Tukey’s pairwise analysis.

### Model development

All models were developed with Python [20] Scikit-learn v0.23.1 and Keras v2.4.0 using the 18 variables in EuroSCORE II - age, gender, renal impairment, extracardiac arteriopathy, poor mobility, previous cardiac surgery, chronic lung disease, active endocarditis, critical preoperative state, diabetes on insulin, New York Heart Association score, Canadian Cardiovascular Society score class 4 angina, left ventricular function, recent myocardial infarction, pulmonary hypertension, operative urgency, weight of the intervention and thoracic aortic surgery [1]. Categorical variables’ values were converted to either binary values for dichotomous categories or to the ordinal scale for multi-category variables.

We acknowledge that the risk profile and outcomes have evolved over time. This is the inherent reason why prediction models need periodic updating. We have temporally split the data for 3 reasons—(i) this mimics the natural development of prediction models with prospective verification of predictive ability following model development, (ii) cohorting the training dataset by time effectively removes the bias of time-based variation in the above predictor and outcome variables when developing the models (i.e. they are all equally effected by temporal changes), (iii) it permitted a review of calibration drift. The 2012–2019 dataset was split with 70% of records (1 January 2012–31 December 2016) for training and 30% (1 January 2017–31 March 2019) for external validation. The ML-models developed were retrained-LR, neural networks (NN), XGBoost and weighted support vector machine (SVM) and random forest (RF) (Supplementary Material, Table S1).

### Model validation

We utilized a modified five-fold cross-validation approach suited to multi-metric assessment with conserved evaluation to extract and vertically combine probabilities and outcome class from each iteration into 2 single vectors (Supplementary Material, Fig. S1). In essence, only 1 model’s prediction is applied to any subset of the validation dataset, thus making the training performance results more comparable to the hold-out dataset.

### Model evaluation

Formulae are provided in Supplementary Material, Table S2.

#### Discrimination

Discrimination is ‘how well the model differentiates those at higher risk of having an event from those at lower risk’ [21, 22]. We are interested in how effectively the models identify those that are likely to experience an event (i.e. death) and so the true positive (TP) rate is of greater significance. The F1 score [23, 24] was used in addition to area under the receiver operator curve (AUC), with the corresponding 95% confidence intervals (CIs) using bootstrap-t sampling with replacement, as it places the emphasis on the TPs and penalizes a high type II error rate [24]. The F1 score is the harmonic mean of the precision and recall and its values range from 0 to 1, with higher scores representing better model performance. 1000 repetitions of normal bootstrap were applied for AUC and 100 repetitions of bootstrap-t were applied for F1 score (Scikit-learn v0.23.1 and ROCR v1.0–11). The bootstrap-t enabled relaxation of parametric assumptions thus reducing computational cost for obtaining a gaussian distribution.

#### Calibration

Calibration is ‘how similar the predicted absolute risk is to the true (observed) risk in groups of patients classified in different risk strata’ [21, 25]. We have included a thorough analysis of model calibration through visual (reliability and residuals curves) and quantitative [expected calibration error (ECE)] [26] means. An ideal model would follow a straight line bisecting the reliability graph with an observed-to-expected ratio (O:E) of 1. A O:E > 1 implies under-estimation of risk. A O:E < 1 implies over-estimation of risk. Higher ECE indicates lower calibration.

Calibration drift was assessed visually by comparing O:E over time and quantified by comparing the ECEs across 2 time periods—2012–2016 and 2017–2019. We assessed models’ calibration by hospital and operation. 95% CIs was derived using bootstrap-t sampling with replacement (100 repetitions).

#### Overall accuracy

The Brier score encompasses both discrimination and calibration. It is the mean squared error between the observed and expected outcomes. 95% CI was derived using bootstrap-t sampling with replacement (100 repetitions). The smaller the Brier score the better the performance of a model.

#### Clinical utility

Assessed using decision curve analysis (DCA) with plotting of net benefit against the threshold probability (p_t_) (defined as the minimum probability of death at which anticipated benefit of surgery equals the anticipated benefit of withholding intervention) [25, 27, 28]. Net benefit was calculated using proportions of TP and false-positive (FP) predictions, weighted by the threshold probability. For comparison, the net benefit when operating on all patients and no patients were plotted at different threshold probabilities. The higher the net benefit for a given threshold, the more clinically useful the model.

Net benefit can be interpreted as:

1. increase in TP and no change in FP—more surgery in those that survive without a change in those that were operated on and died—i.e. more surgery in survivors
2. no change in TP and decrease in FP—less surgery in those that would die and no change in surgery on those that survived—i.e. less surgery in the non-survivors.

### Variable importance over time

Trends were assessed for 2012–2016 and 2017–2019 independently. Variable importance for retrained-LR and SVM is based on weight coefficients. XGBoost is based on gain and RF is based on Gini importance. NN were assessed using variance-based feature importance [29]. The baselines were generated by the mean of each variable scaled to values between 0 and 1 using min–max normalization. The analysis was performed using five-fold cross-validation and the mean plotted. 95% CI was derived using bootstrap-t sampling with replacement (100 repetitions).

## RESULTS

### Demographics

**Figure 1:**
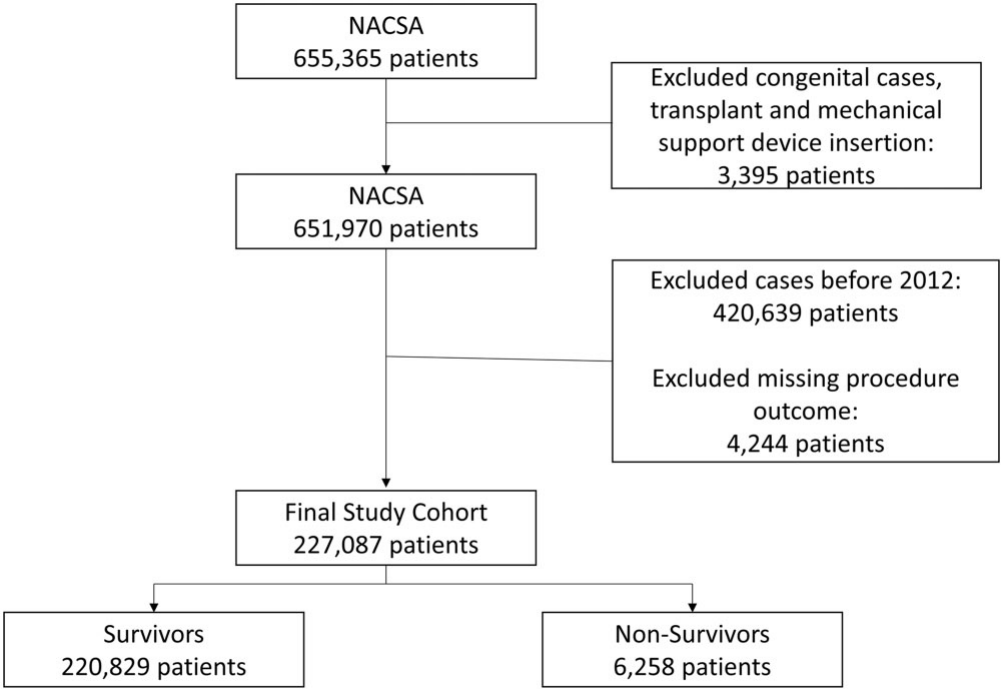
Consort diagram showing flow of participants through the study.

A total of 227 087 adults underwent cardiac surgery during the study period. Congenital, transplant and mechanical support device insertion cases were excluded. The pre-processing of data has been described previously [17] and is in accordance with previously published guidance [18]. Missing data were <1%. There were 6258 deaths (2.76%) (Fig. 1). Baseline differences in EuroSCORE II variables between survivors and non-survivors are shown in Table 1.

**Table 1:** Baseline patient demographics

Variable	Mortality status		*P*-Value^b^
	Survivor, *N* = 220 829^a^	Non-survivor, *N* = 6258^a^	
EuroSCOREII, mean (SD)	0.03 (0.04)	0.12 (0.14)	<0.001
Age (years), mean (SD)	67.5 (11.2)	70.8 (11.4)	<0.001
Female gender, *n* (%)	59 467 (27%)	2328 (37%)	<0.001
Diabetes on insulin, *n* (%)	12 818 (5.8%)	453 (7.2%)	<0.001
Recent myocardial infarct, *n* (%)	43 316 (20%)	1594 (25%)	<0.001
New York Heart Association score, *n* (%)			<0.001
I	48 625 (22%)	1055 (17%)	
II	96 888 (44%)	1609 (26%)	
III	64 049 (29%)	2228 (36%)	
IV	11 267 (5.1%)	1366 (22%)	
Renal impairment, *n* (%)			<0.001
Normal (creatinine clearance > 85 ml/min)	103 196 (47%)	1704 (27%)	
Moderate (50 ml/min < creatinine clearance < 85 ml/min)	92 411 (42%)	2451 (39%)	
Severe (creatinine clearance < 50 ml/min)	23 035 (10%)	1773 (28%)	
On dialysis	2187 (1.0%)	330 (5.3%)	
Chronic lung disease, *n* (%)	26 644 (12%)	1211 (19%)	<0.001
Poor mobility, *n* (%)	8305 (3.8%)	514 (8.2%)	<0.001
Extracardiac arteriopathy, *n* (%)	22 327 (10%)	1215 (19%)	<0.001
Previous cardiac surgery, *n* (%)	12 012 (5.4%)	1141 (18%)	<0.001
Left ventricular function (LVEF: left ventricular ejection fraction)			<0.001
Good (LVEF > 50%)	184 721 (84%)	4706 (75%)	
Moderate (LVEF 31–50%)	30 608 (14%)	1089 (17%)	
Poor (LVEF 21–30%)	4241 (1.9%)	318 (5.1%)	
Very poor (LVEF ≤ 20%)	1259 (0.6%)	145 (2.3%)	
Pulmonary hypertension, *n* (%)			<0.001
Pulmonary artery systolic pressure (<31 mmHg)	201 643 (91%)	5000 (80%)	
Pulmonary artery systolic pressure (31–55 mmHg)	13 126 (5.9%)	705 (11%)	
Pulmonary artery systolic pressure (>55 mmHg)	6060 (2.7%)	553 (8.8%)	
Canadian cardiovascular society score 4, *n* (%)	18 370 (8.3%)	956 (15%)	<0.001
Operative urgency, *n* (%)			<0.001
Elective	141 617 (64%)	2442 (39%)	
Urgent	72 090 (33%)	2134 (34%)	
Emergency	6533 (3.0%)	1230 (20%)	
Salvage	589 (0.3%)	452 (7.2%)	
Weight of the intervention, *n* (%)			<0.001
Isolated CABG	111 243 (50%)	1546 (25%)	
Single non-CABG	62 568 (28%)	2153 (34%)	
Two procedures	42 649 (19%)	2108 (34%)	
Three procedures	4369 (2.0%)	451 (7.2%)	
Critical preoperative state, *n* (%)	7255 (3.3%)	1382 (22%)	<0.001
Active endocarditis, *n* (%)	5816 (2.6%)	493 (7.9%)	<0.001
Surgery on thoracic aorta, *n* (%)	9070 (4.1%)	896 (14%)	<0.001

a Mean (SD) or frequency (%).

b Wilcoxon rank sum test; Pearson’s Chi-squared test.

CABG, coronary artery bypass graft; EuroSCORE: European System for Cardiac Operative Risk Evaluation; LVEF, left ventricular ejection fraction.

### Model performance

**Figure 2:**
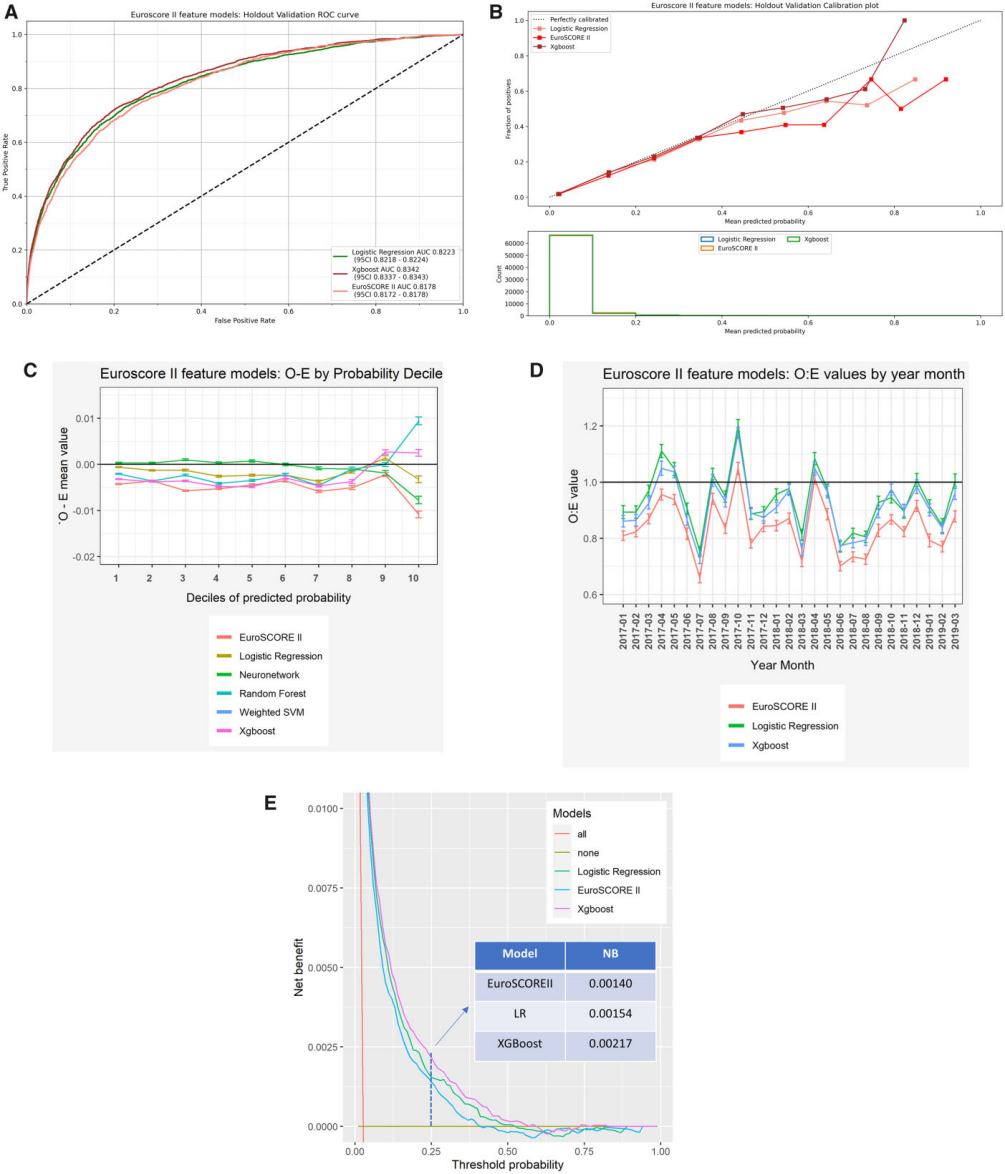
Assessment of model performance. (**A**) Discrimination—area under the receiver operator curve (AUC) of different prediction models. (**B**) Calibration—ratios of observed (O)-to-expected (E) outcomes per decile of predicted risk. (**C**) Calibration—difference in observed (O) and expected (E) outcomes per decile of predicted risk. (**D**) Assessment of calibration drift. (**E**) Decision curve analysis showing the expected net benefit of performing surgery on all patients, no patients and patients stratified by retrained-logistic regression, EuroSCORE II and XGBoost at different probability thresholds.

All models displayed good discrimination with all values of AUC > 0.81. Ensemble decision tree-based algorithms, namely XGBoost and RF, consistently out-performed other models (Table 2, Fig. 2A, and Supplementary Material, Fig. S2A). The AUC for XGBoost and RF were significantly greater than those for EuroSCORE II and retrained-LR (Table 2). Similarly, F1 scores for XGBoost and RF were greater than those for EuroSCORE II and retrained-LR. EuroSCORE II had the lowest mean AUC and F1 of all models assessed in the training and validation subsets (Supplementary Material, Fig. S2A).

**Table 2:** Quantitative model performance

Model name	Discrimination		Calibration	Brier score^a^
	AUC^a^	F1^a^	ECE^a^ (×10^–3^)	
(A) Training dataset				
XGBoost	**0.826** (0.825–0.826)	**0.267** (0.266–0.269)	3.22 (3.14–3.29)	**0.0245** (0.024–0.0246)
RF	0.824 (0.824–0.824)	0.266 (0.265–0.267)	3.30 (3.23–3.37)	0.0246 (0.0245–0.0247)
Retrained-LR	0.818 (0.818–0.818)	0.259 (0.258–0.261)	**1.73** (1.67–1.79)	0.0248 (0.0247–0.0248)
NN	0.817 (0.816–0.817)	0.252 (0.251–0.253)	2.12 (2.06–2.19)	0.0248 (0.0247–0.0248)
EuroSCORE II	*0.814* (0.814–0.814)	*0.244* (0.243–0.245)	3.92 (3.85–3.99)	0.0250 (0.0249–0.0250)
Weighted SVM	0.818 (0.818–0.819)	0.252 (0.250–0.253)	*192.02* (191.94–192.11)	*0.0709* (0.0709–0.0710)
(B) Validation dataset				
XGBoost	**0.834** (0.834–0.834)	0.278 (0.276–0.280)	3.94 (3.85–4.03)	**0.0236** (0.0235–0.0237)
RF	0.834 (0.833–0.834)	**0.279** (0.277–0.281)	3.56 (3.46–3.66)	0.0236 (0.0235–0.0237)
Retrained-LR	0.822 (0.822–0.822)	0.266 (0.264–0.268)	2.34 (2.25–2.43)	0.0237 (0.0236–0.0238)
NN	0.820 (0.812–0.820)	0.272 (0.270–0.274)	**1.92** (1.82–2.02)	0.0238 (0.0237–0.0239)
EuroSCORE II	*0.818* (0.817–0.818)	*0.253* (0.258–0.262)	5.20 (5.08–5.32)	0.0241 (0.0240–0.0242)
Weighted SVM	0.823 (0.823–0.823)	0.260 (0.252–0.255)	*203.51* (203.36–203.66)	*0.0749* (0.0748–0.0750)

Best performing models are highlighted in bold and worst performing models are in italics.

a Mean (95% confidence interval).

AUC, area under the receiver operator curve; ECE, expected calibration error; EuroSCORE, European System for Cardiac Operative Risk Evaluation; LR, logistic regression; NN, neural networks; RF, random forest; SVM, support vector machine.

Retrained-LR and NN had the lowest ECEs in the training and validation datasets, respectively (Table 2). SVM had the highest ECE in both subsets, followed by EuroSCORE II. Pairwise comparisons (Supplementary Material, Table S3) showed that all models displayed better calibration than EuroSCORE II, except SVM.

Reliability graphs (Fig. 2B and Supplementary Material, Fig. S2B) showed that all models had performances close to the ‘ideal’ bisector line at predicted risk under 25%. Between 25% and 45% predicted risk, RF underestimated mortality risk. Above 35% predicted risk, other models slightly overestimated risk. Of note, most patients had a predicted probability of death of <10% (Fig. 2B). All models showed a tendency towards overestimation with time (Fig. 2D) although the calibration drift was lower in NN, XGBoost and RF compared with EuroSCORE II. EuroSCORE II overestimated risk across all deciles of risk and over time (Fig. 2C and D). Other models marginally overestimated risk in all but the last decile.

The best performing model as per the Brier score was XGBoost followed by RF (Table 2). SVM and EuroSCORE II had significantly poorer performances than other models.

DCA (Fig. 2E and Supplementary Material, Fig. S2C) showed XGBoost had the greatest net benefit for threshold probabilities ≤0.5, exceeding that of both EuroSCORE II and retrained-LR. At a threshold probability of 25%, the net benefit of XGBoost (0.00217) was 0.0008 greater than EuroSCORE II (0.00140) and 0.0006 greater than retrained-LR (0.00154), equating to a net benefit of 24 per 10 000 patients (545 patients in our cohort) when using XGBoost compared to EuroSCORE II. The net benefit is 16 per 10 000 people (409 patients in our cohort) when using XGBoost compared to retrained-LR. Above the 0.5 threshold, there is an overlap of the curves, as would be expected at such probabilities of risk.

**Figure 3:**
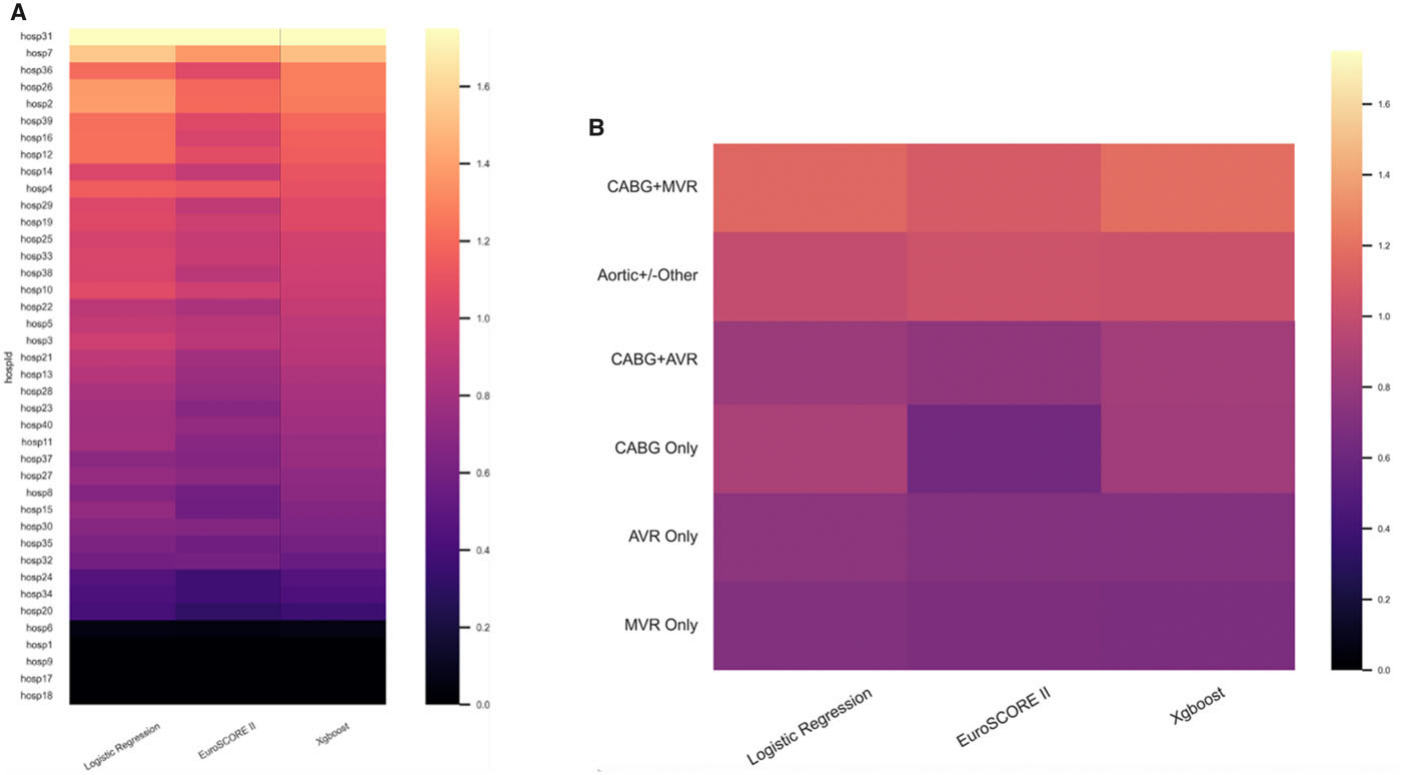
Variations in calibration (observed-to-expected ratio) across hospitals (**A**) and operations (**B**) by different models. Ranked by observed-to-expected ratio for the best performing model (XGBoost).

Interestingly, there was marked variation in model calibration across different hospitals (Fig. 3A) and different operations (Fig. 3B). Within a hospital, there was little change in calibration using different models suggesting that local hospital practices or demographic factors may be unaccounted for by changes in model development. Surprisingly, EuroSCORE II over-estimates risk for isolated coronary artery bypass graft (CABG), aortic valve replacement (AVR) and mitral valve repair/replacement (MVR). Retrained-LR and XGBoost over-estimate risk in isolated AVR and MVR but have comparatively better performance in isolated CABGs. XGBoost more accurately predicts mortality following CABG + AVR compared to EuroSCORE II and retrained-LR. EuroSCORE II and XGBoost have greater accuracy for aortic procedures compared to retained-LR. CABG + MVR risk is under-estimated by XGBoost and LR, with greater accuracy using EuroSCORE II. Detailed analysis of operation-based model performance is being conducted by our group.

### Variable importance

**Figure 4:**
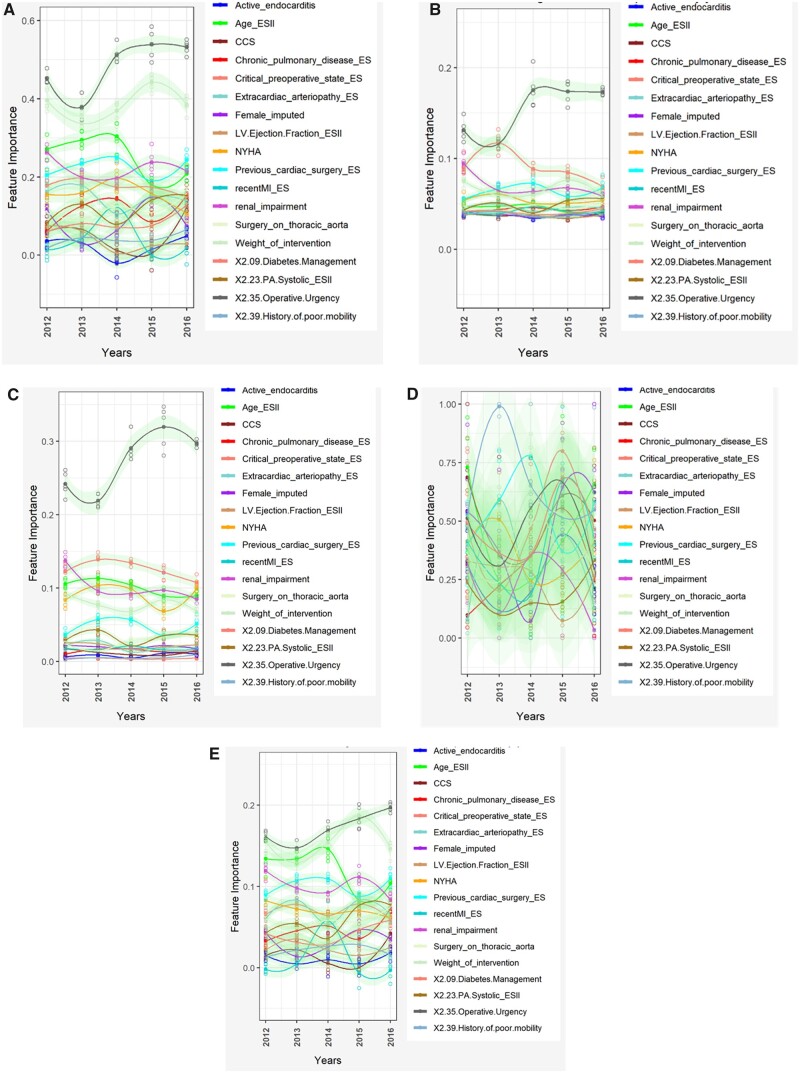
Variable importance in different models over time. (**A**) Logistic regression (retrained). (**B**) XGBoost. (**C**) Random forest. (**D**) Neural network. (**E**) Weighted support vector machine.

Over time, there were subtle changes in variable importance (Fig. 4 and Supplementary Material, Fig. S3). Most models showed operative urgency had the most significant influence, with a sharp increase in importance between 2012 and 2014 and plateau thereafter. The significance of age decreased from 2014 onwards for EuroSCORE II, RF, SVM and XGBoost. New York Heart Association score was of greater importance in RF and XGBoost. The influence of renal impairment increased somewhat in EuroSCORE II but conversely decreased in RF and XGBoost. NN variable-importance had the most marked swings over time and no consistent trends could be identified. XGBoost displayed the most subtle changes. SVM and EuroSCORE II had similar variable-importance profiles.

## DISCUSSION

This is the largest multicentre comparative analysis of ML-models in adult patients undergoing cardiac surgery. The primary finding is that ML-models improve upon the discrimination and clinical utility of EuroSCORE II. There were subtle improvements in calibration, which were similar to retrained-LR. This is in contrast to our previous single-centre results [16]. Notably these improvements were found utilizing the same limited number of variables as EuroSCORE II. Our group will apply feature selection techniques to the entire dataset to search for additional risk factors that could be included in future models.

The increased cohort size has contributed to more accurate model development, as is evidenced by greater AUC across all models. The best performing ML-model showed a 1.6% improvement in discrimination, that nationally would equate to hundreds of patients being incorrectly counselled and denied surgery. Ensemble decision tree-based models (XGBoost and RF) showed greatest discrimination. XGBoost had the best overall performance using the Brier score.

Pairwise quantitative comparisons showed NN and retrained-LR had the highest calibration, followed by RF and XGBoost. Calibration drift was least pronounced for NN and XGBoost. Periodic retraining of models is accepted in clinical practice and indeed the Society of Thoracic Surgeons does so on an annual basis. Periodic automated retraining of models may be possible in the future as server capacity increases. Reliability graphs showed good calibration of all models at predicted risk < 35% with a separation of the curves thereafter. However, this is a cohort of patients that have proceeded to have surgery and, as expected, most had predicted mortality <10% and almost none had a predicted mortality > 50%. With very few high-risk cases, the models are insufficiently trained on this cohort resulting in lower calibration. As some high-risk patients did undergo surgery, factors not accounted for in the database may balance the risk perceived by the current variables. The ‘end-of-bed’ inspection adds a layer of clinic judgement that is difficult incorporate in a rigid database. Additional information from clinicians and inclusion of additional clinical and radiological information is needed in future models. Many risk factors have previously been identified [30] but gathering such vast quantities of data are logistically challenging.

The differences in AUC, F1 score and ECE show statistical significance. However, in an over-powered study, one must interpret the results with clinical context. Hence, we reviewed clinical utility using DCA and found that the greatest net benefit from treatment was achieved using XGBoost and would result in many more patients appropriately being offered surgery as compared to EuroSCORE II. Given that the mortality rate was low (2.76%), seemingly small statistically significant improvements may also be of clinical significance. This corroborated previous findings [27].

The present literature has shown conflicting results for the use of ML-models in mortality risk prediction in adult cardiac surgery. Some have shown no improvement in discrimination [16], whilst others only modest improvements [14, 15, 27]. Few have reported calibration [15, 16] and only 1 reported clinical utility [27]. Current research into ensemble models, super-learners and stacked models show improved model performance [15]. Our future work will focus on ensemble modelling.

ML-models are often described as the ‘black box’ of risk prediction and this dissuades clinicians from trusting their results. We attempted to demystify this by identifying the underlying variable importance. Different strategies were employed to reflect the different mechanisms for model development and results regularized to allow cross-model comparisons. Operative urgency had the greatest influence on risk, which is both intuitive and consistent with previous knowledge [1]. Notably, age had a decreasing contribution to risk. Further analysis of trends in prevalence of predictor variables over time would be useful.

Another criticism of risk modelling is the cohort-specific interpretability of the results. We noted hospital and operation-specific variations in model calibration. Conspicuously, model calibration is specific to a hospital or procedure rather than the model employed. This suggests that local factors have not been accounted for in current models. Automated data extraction from electronic health records (EHR), use of natural language processing for report extraction, image analysis and greater information on local health provision could potentially be incorporated into future models to increase accuracy and applicability. This would necessitate accounting for highly complex interactions and prove computationally expensive when using LR. ML-models may therefore be best equipped to tackle this challenge in the future. Conversely an institution, operation or surgeon-specific risk adjusted mortality rate may need to be utilized when counselling patients or deciding local policies.

Our study supports the study of ML-models in future iterations of risk-stratification models and has provided a robust set of metrics to test future models’ performance.

### Limitations

Only 3 months of data for 2019 were available and future efforts to obtain a more up-to-date dataset are planned. We also plan to perform a dedicated study on the optimal performance hyperparameters and configurations for NN on the current dataset. We will be interested in assessing stability of post-pandemic risk prediction based on pre-pandemic data. Class imbalance does exist within the dataset and can be tackled with different strategies such as over and under-sampling or algorithm-centred approaches that modify the algorithm to favour its prediction towards the less-represented class. However, these approaches are controversial and have not been employed in this analysis.

## CONCLUSIONS

Machine learning models showed some statistical improvements in discrimination, calibration and clinical utility compared with EuroSCORE II. All models showed a degree of calibration drift. Ensemble decision tree-based models showed the most accurate performance. Overall, operative urgency remains the primary contributor to mortality risk prediction. The clinical impact of these improvements are modest at present. However, the incorporation of additional risk factors, including the use of imaging analysis and natural language processing, in future studies may improve upon these findings and warrants further study. Furthermore, the prospect of automated retraining of models would be beneficial in reducing calibration drift.

## Supplementary Material

ezad183_Supplementary_Data

## Data Availability

The data underlying this article were provided by National Institute of Cardiovascular Outcomes Research with permission from HQIP/HRA. Data will be shared on request to the corresponding author with permission from HQIP/HRA.

## ABBREVIATIONS

AUC: Area under the receiver operator curve
CI: Confidence interval
E: Expected outcome
EuroSCORE: European System for Cardiac Operative Risk Evaluation
FP: False positive
LR: Logistic regression
NN: Neural network
O: Observed outcome
RF: Random forest
SVM: Support vector machine
TP: True positive
