# Corrigendum to: Comparison of machine learning techniques in prediction of mortality following cardiac surgery: analysis of over 220 000 patients from a large national database

**DOI:** 10.1093/ejcts/ezad370

**Published:** 2023-11-14

This is a corrigendum to: Shubhra Sinha, Tim Dong, Arnaldo Dimagli, Hunaid A Vohra, Chris Holmes, Umberto Benedetto, Gianni D Angelini, Comparison of machine learning techniques in prediction of mortality following cardiac surgery: analysis of over 220 000 patients from a large national database, European Journal of Cardio-Thoracic Surgery, Volume 63, Issue 6, June 2023, ezad183, https://doi.org/10.1093/ejcts/ezad183

In the originally published version of this manuscript, BHF funder grant number SP/19/7/34810 was omitted.

This has been corrected.

