# Supplementary material for: Comparison of machine learning techniques in prediction of mortality following cardiac surgery: analysis of over 220 000 patients from a large national database
Source: Eur J Cardiothorac Surg. 2023 May 8;63(6):ezad183. doi: 10.1093/ejcts/ezad183 (PMC10275911; doi:10.1093/ejcts/ezad183)

**Supplemental Material**

**Comparison of Machine Learning Techniques in Prediction of Mortality following Cardiac Surgery: Analysis of over 220,000 patients from a Large National Database**

*Shubhra Sinha MBBS, Tim Dong MSc, Arnaldo Dimagli MD, Hunaid A Vohra PhD, Chris Holmes PhD, Umberto Benedetto PhD and Gianni D Angelini MD.*

References:

1. Head SJ, Osnabrugge RLJ, Howell NJ, Freemantle N, Bridgewater B, Pagano D, et al. A systematic review of risk prediction in adult cardiac surgery: Considerations for future model development. Eur J Cardio-thoracic Surg. 2013;43(5).

32. Lippmann RP, Shahian DM. Coronary artery bypass risk prediction using neural networks. Ann Thorac Surg. 1997;63(6):1635–43.

33. Tu J V., Weinstein MC, McNeil BJ, Naylor CD. Predicting mortality after coronary artery bypass surgery: What do artificial neural networks learn? Med Decis Mak. 1998;18(2):229–35.

34. Christodoulou E, Ma J, Collins GS, Steyerberg EW, Verbakel JY, Van Calster B. A systematic review shows no performance benefit of machine learning over logistic regression for clinical prediction models. J Clin Epidemiol [Internet]. 2019;110:12–22. Available from: https://doi.org/10.1016/j.jclinepi.2019.02.004

35. Buzatu DA, Taylor KK, Peret DC, Darsey JA, Lang NP. The determination of cardiac surgical risk using artificial neural networks. J Surg Res. 2001;95(1):61–6.

36. Ghavidel AA, Javadikasgari H, Maleki M, Karbassi A, Omrani G, Noohi F. Two new mathematical models for prediction of early mortality risk in coronary artery bypass graft surgery. J Thorac Cardiovasc Surg [Internet]. 2014;148(4):1291-1298.e1. Available from: http://dx.doi.org/10.1016/j.jtcvs.2014.02.028

37. Wong J et al., Manderson T, Abrahamowicz M, Buckeridge DL, Tamblyn R. Can Hyperparameter Tuning Improve the Performance of a Super Learner? . Epidemiol . 2019;30(4):521–31.

38. Ali L, Niamat A, Khan JA, Golilarz NA, Xingzhong X, Noor A, et al. An Optimized Stacked Support Vector Machines Based Expert System for the Effective Prediction of Heart Failure. IEEE Access. 2019;7:54007–14.

39. Elshawi R, Al-Mallah MH, Sakr S. On the interpretability of machine learning-based model for predicting hypertension. BMC Med Inform Decis Mak. 2019;19(1).

40. Celi LA, Galvin S, Davidzon G, Lee J, Scott D, Mark R. A database-driven decision support system: Customized mortality prediction. J Pers Med. 2012;2(4):138–48.

Supplementary Table 1. Machine learning models developed with final parameters.

| **Model** | **Description** | **Parameters** |
| --- | --- | --- |
| Retrained Logistic Regression | EuroSCOREII risk factors were fitted with an LR(retrained LR) model. Regularization reduces the risk of over-fitting. Inverse of regularization strength set to default value of 1, with smaller values resulting in stronger regularization and vice versa. | Inverse of regularization strength(c)=1; Maximum iterations=100 |
|  |  |  |
| Neural Network | A form of deep learning where information is processed via a method that mimics how organic nervous systems process information. The input layer consists of predictor/input variables, which are prescribed different weights and processed via a series of interconnected layers of neurons/hidden layers till an output value/layer is reached. This is compared to the true output and an error calculated. The weights ascribed to the input variables are then altered till this error is minimised. NN was trained using 1000 epochs, with batch size of 20,000. The best model was saved using early stopping to reduce likelihood of overfitting(20). NN are computationally costly (time and computer resources) to tune for large datasets. Thus, we have used a subpopulation of the entire dataset(from our previous study(16)) to tune and identify the optimal hyperparameters to use and expect that this would be a sufficient approximation of the true hyperparameters of the entire dataset. | Adam optimizer; Binary cross-entropy loss function; input layer n = 18 nodes, hidden layer one n = 90 nodes, hidden layer two n = 36 nodes and output layer one node |
|  |  |  |
| XGBoost | An ensemble model created from multiple iterations of weaker prediction models, typically short decision trees. With each iteration the XGBoost model determines the residual difference between its prediction and the true value and a weak decision tree is modelled to predict this residual. The weak tree is them added to the ensemble and weighted in accordance with its prediction accuracy. Thus it a computationally more costly but robust model. 3-fold Grid Search Cross Validation was applied using 2012-2016 dataset | Maximum depth= 5, Step size shrinkage= 1, objective= binary=logistic, Subsample ratio of the training instances= 0.6, Minimum sum of instance weight (hessian) needed in a child= 1, Minimum loss reduction= 0.5, Subsample ratio of columns when constructing each tree= 0.8, learning_rate= 0.02, n_estimators = 300 |
| Weighted Support Vector Machine | Plots training examples to points in space to create a hyperplane that maximises the width between classes. New examples are mapped to the same space and depending on their position relative to the hyperplane their class is predicted. 3-fold Grid Search Cross Validation using 2012-2016 dataset. | C=0.01, class_weight=balanced, dual=False, tol=1e-05 |
| Random Forest | Decision trees are non-linear models that look like a flow chart. A condition is used to determine how to split the data to result in greatest homogeny of the subsets produced. Each division if known as a branch. Branching continues till the final decision is complete and the output/leaf node determines the model’s prediction. The model can be fine-tuned by pre-determining the depth of the model and ‘pruning’ those branches that have low importance. We manually tuned parameters in response to model discrimination(AUC) evaluated with cross-validation | Estimators n = 700, maximum depth n = 10, minimum samples split n = 5, minimum samples leaf n = 20 |
|  |  |  |

Supplementary Table 2. Formulae for metrics of model performance.

| Metric | Formula |
| --- | --- |
| F1 score | $F1=\frac{2*PPV*TPR}{PPV+TPR}$  PPV=positive predictive value. TPR=true positive rate |
| Expected Calibration Error | $ECE= \sum_{i=1}^{k} P_{i}*(O_{i}- E_{i})$  O=observed outcome; E=expected or predicted outcome; k=number of bins; P(i)=proportion of all patients in bin i |
| Brier Score | $Brier Score= \frac{{\sum_{i=1}^{N} {(E}_{i}-O_{i})}^{2}}{N}$  O=observed outcome; E=expected or predicted outcome; N=sample size |
| Net Benefit | $Net Benefit=\frac{TP}{N}-\frac{FP}{N}\left( \frac{p_{t}}{1-p_{t}} \right)$  TP-true positive;FP-false positive; $p_{t}:probability threshold$ |

Supplementary Table 3. Tukey’s pairwise comparisons of calibration, as measured by the expected calibration error, in the validation subset.

| **Model 1** | **Model 2** | **Difference in ECE** | **95% Confidence Interval** | **Adjusted p-value** |
| --- | --- | --- | --- | --- |
| LR | EuroSCOREII | -0.0029 | (-0.0031, -0.0026) | p<0.0001 |
| XGBoost | EuroSCOREII | -0.0013 | (-0.0015, -0.001) | p<0.0001 |
| RF | EuroSCOREII | -0.0016 | (-0.0019, -0.0014) | p<0.0001 |
| NN | EuroSCOREII | -0.0033 | (-0.0035, -0.0031) | p<0.0001 |
| wSVM | EuroSCOREII | 0.1983 | (0.1981, 0.1985) | p<0.0001 |
| XGBoost | LR | 0.0016 | (0.0014, 0.0018) | p<0.0001 |
| RF | LR | 0.0012 | (0.0010, 0.0014) | p<0.0001 |
| NN | LR | -0.0004 | (-0.0006, -0.0002) | p<0.0001 |
| wSVM | LR | 0.2012 | (0.2010, 0.2014) | p<0.0001 |
| XGBoost | RF | 0.0004 | (0.0002, 0.0006) | p<0.0001 |
| XGBoost | NN | 0.0020 | (0.0018, 0.0022) | p<0.0001 |
| RF | NN | 0.0016 | (0.0014, 0.0019) | p<0.0001 |
| XGBoost | wSVM | -0.1996 | (-0.1998, -0.1994) | p<0.0001 |
| wSVM | RF | 0.2000 | (0.1997, 0.2002) | p<0.0001 |
| wSVM | NN | 0.2016 | (0.2014, 0.2018) | p<0.0001 |

ECE: Expected calibration error, LR: Logistic Regression, EuroSCORE: European System for Cardiac Operative Risk Evaluation, RF: Random forest, NN: Neural network, wSVM: Weighted support vector machine

Supplementary Figure 1. Explanation of model development using concatenation of outputs following 5-fold cross-validation.

1. Typical Risk Model Evaluation Method use ensemble-like performance evaluation and can lead to overconfident and more stable prediction evaluation than that would be expected in the hold-out datasets

Typical existing cross validation packages/libraries for cross validation such as CalibratedClassifierCV currently fits and trains an estimator for each fold. All the estimators fit at each fold are kept in a list. At prediction time, every estimator makes a prediction, and the **average** of the returned prediction probabilities (or sometimes in other packages the average of the AUC) is the final prediction. This typically makes model performance better (more confident/generalizable) than the results from each fold individually, giving an ensemble-like performance evaluation. This often results in overconfidence of the model performance and a more stable prediction than that would be expected in the hold-out datasets.

**
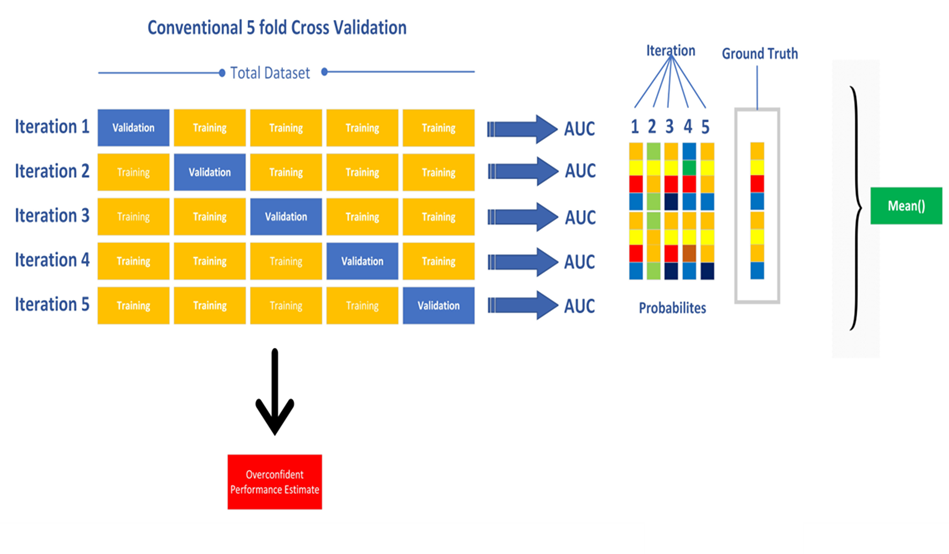
**

1. Modified Method using conserved performance evaluation that removes ensemble-like averaging effects in the evaluation process

In our approach, we vertically concatenate the prediction probabilities and the ground truth outcome labels from each cross-validation iteration. So, in essence only one model’s prediction is applied to any subset of the validation dataset, rather than having 5 models predicting multiple times on the validation dataset and averaging performance. The results from our approach is therefore a more realistic and conserved estimate of the training dataset performance. This also makes the training performance results more comparable to the hold-out dataset, which does not use cross validation.
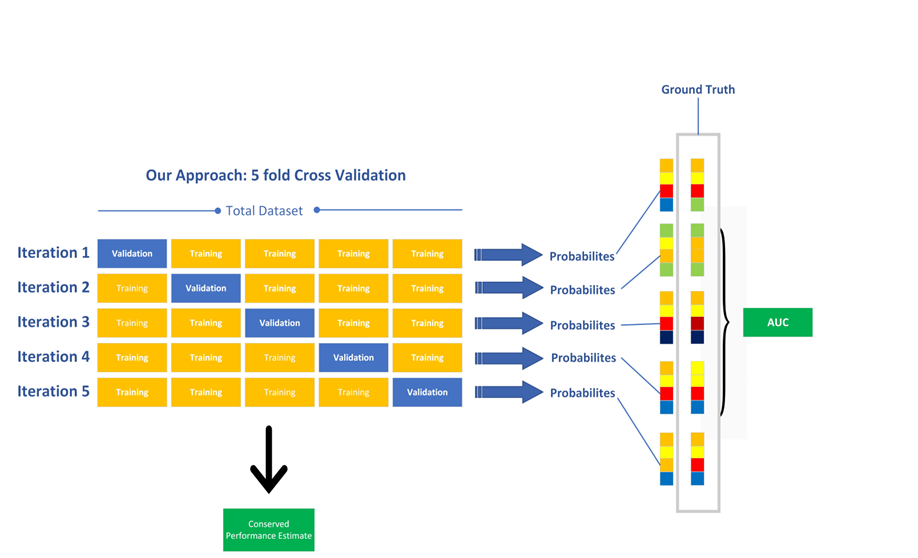


The training model used in effect only 4/5^th^ of the training data to predict each subset of validation folds data at any one time (and there is no ensemble-like averaging as in the conventional approach), whilst the models for the hold-out set had a large availability of training data i.e., the entire training data (5/5). Thus, the hold-out models performed better than the cross-validation models on the training data.

Supplementary Figure 2. Assessment of Model Performance.

A) Discrimination - Area under the receiver operator curve (AUC) of different prediction models.


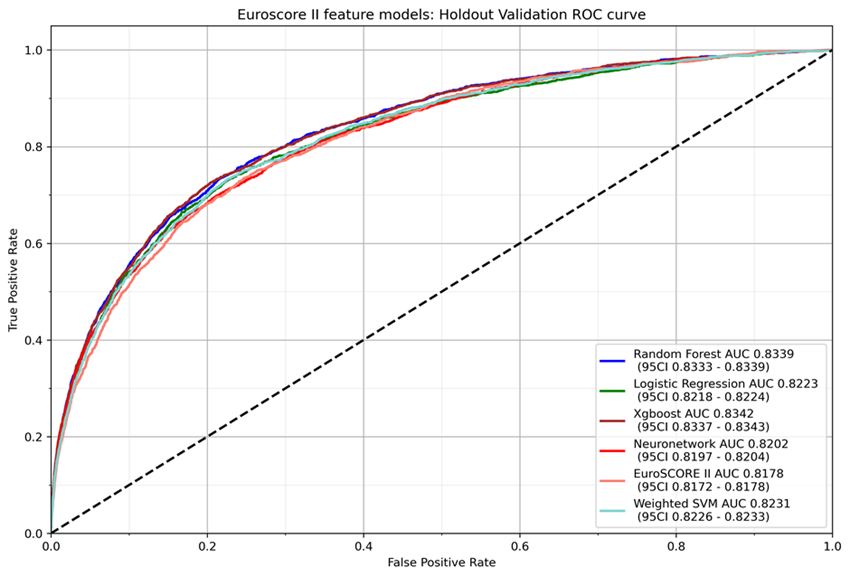


B) Calibration – Ratios of observed and expected outcomes per decile of predicted risk.


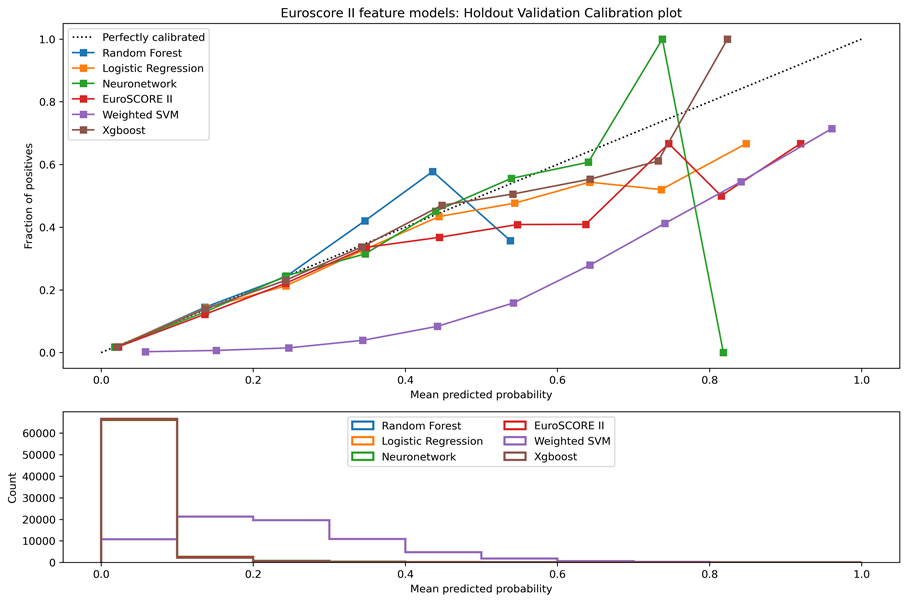


1. Decision curve analysis showing the expected net benefit of performing surgery on all patients (red), no patients (green), patients stratified by model at different probability thresholds.


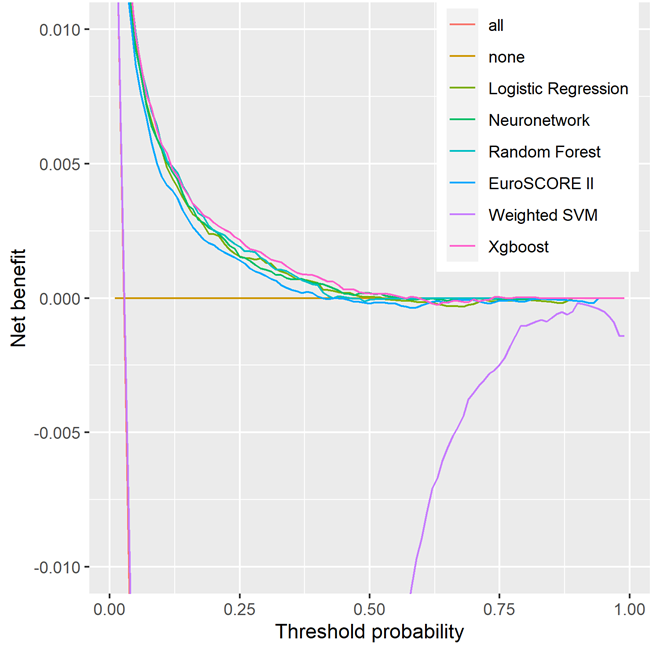


Supplementary Figure 3. Variable importance change over time by model. We generated all model feature importance over time in one plot and with the mean values of each feature as baselines.


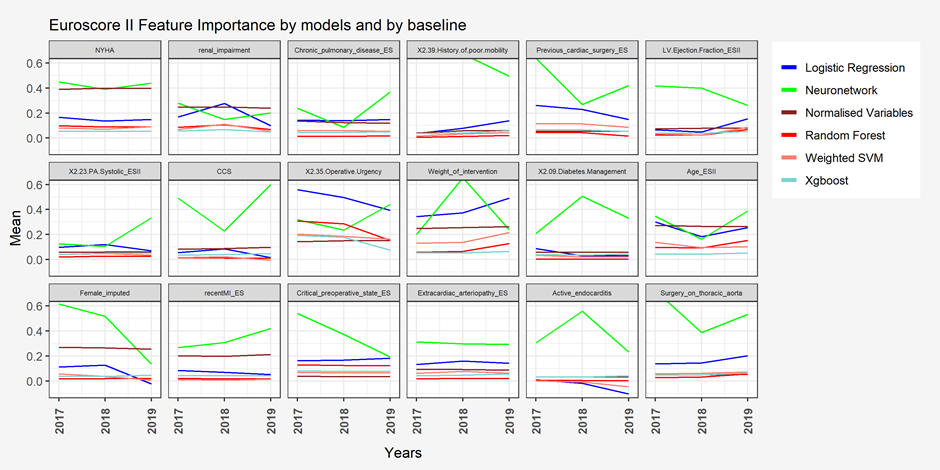

Supplement: ezad183_Supplementary_Data [file ezad183_supplementary_data.docx]
